# A *de novo *transcriptome of the Asian tiger mosquito, *Aedes albopictus*, to identify candidate transcripts for diapause preparation

**DOI:** 10.1186/1471-2164-12-619

**Published:** 2011-12-20

**Authors:** Monica F Poelchau, Julie A Reynolds, David L Denlinger, Christine G Elsik, Peter A Armbruster

**Affiliations:** 1 Department of Biology, Georgetown University, 37th and O Streets NW, Washington, DC, USA; 2 Department of Entomology, Ohio State University, 318 W 12th Ave., Columbus, Ohio, USA

## Abstract

**Background:**

Many temperate insects survive the harsh conditions of winter by undergoing photoperiodic diapause, a pre-programmed developmental arrest initiated by short day lengths. Despite the well-established ecological significance of photoperiodic diapause, the molecular basis of this crucial adaptation remains largely unresolved. The Asian tiger mosquito, *Aedes albopictus *(Skuse), represents an outstanding emerging model to investigate the molecular basis of photoperiodic diapause in a well-defined ecological and evolutionary context. *Ae. albopictus *is a medically significant vector and is currently considered the most invasive mosquito in the world. Traits related to diapause appear to be important factors contributing to the rapid spread of this mosquito. To generate novel sequence information for this species, as well as to discover transcripts involved in diapause preparation, we sequenced the transcriptome of *Ae. albopictus *oocytes destined to become diapausing or non-diapausing pharate larvae.

**Results:**

454 GS-FLX transcriptome sequencing yielded >1.1 million quality-filtered reads, which we assembled into 69,474 contigs (N50 = 1,009 bp). Our contig filtering approach, where we took advantage of strong sequence similarity to the fully sequenced genome of *Aedes aegypti*, as well as other reference organisms, resulted in 11,561 high-quality, conservative ESTs. Differential expression estimates based on normalized read counts revealed 57 genes with higher expression, and 257 with lower expression under diapause-inducing conditions. Analysis of expression by qPCR for 47 of these genes indicated a high correlation of expression levels between 454 sequence data and qPCR, but congruence of statistically significant differential expression was low. Seven genes identified as differentially expressed based on qPCR have putative functions that are consistent with the insect diapause syndrome; three genes have unknown function and represent novel candidates for the transcriptional basis of diapause.

**Conclusions:**

Our transcriptome database provides a rich resource for the comparative genomics and functional genetics of *Ae. albopictus*, an invasive and medically important mosquito. Additionally, the identification of differentially expressed transcripts related to diapause enriches the limited knowledge base for the molecular basis of insect diapause, in particular for the preparatory stage. Finally, our analysis illustrates a useful approach that draws from a closely related reference genome to generate high-confidence ESTs in a non-model organism.

## Background

The annual arrival of winter in temperate habitats represents a fundamental challenge to the survival and reproduction of a wide variety of insects. Many temperate insects surmount the harsh conditions of winter by undergoing photoperiodic diapause, a process in which day length (photoperiod) provides a token cue that initiates a pre-programmed and hormonally controlled developmental arrest in advance of the onset of unfavorable conditions [reviewed in [1]]. Photoperiodic diapause is thus a crucial ecological adaptation enabling temperate insects to coordinate growth, development, reproduction and dormancy in a seasonal environment. Processes related to regulation of development, metabolic depression, stress tolerance and nutrient storage appear to be particularly important physiological components of the diapause response [2-8].

While many aspects of the physiological and ecological controls of diapause are known, research on the molecular bases of diapause has been hampered by the lack of genetic information from a suitable model organism [9]. *Drosophila melanogaster *has a weak diapause response that is highly temperature-dependent [10], and thus can give only limited insight into the mechanistic basis of photoperiodic diapause [11]. *Bombyx mori *provides a rich source of information on gene expression during diapause. However, diapause entry in *B. mori *is controlled by diapause hormone, which is poorly conserved throughout insects [12], and is thus of limited use for comparative analyses. Recently, the advent of high-throughput sequencing methods has facilitated genetic and genomic analyses of life-history traits in non-model systems [5,13,14]. These new technologies allow for *de novo *characterization of genome-wide expression in non-model organisms, and have already led to exciting recent progress on the transcriptional bases of diapause in several insect taxa [3,5,15-17].

An additional factor that has limited progress on understanding the molecular bases of diapause is the wide diversity of diapause syndromes among different insect species. The diapause program is characterized by three eco-physiological phases: pre-diapause, diapause, and post-diapause [18]. During the pre-diapause phase the individual is sensitive to token environmental cue(s) and in response to appropriate stimuli will initiate preparation for entry into diapause. During the diapause phase metabolism is reduced and direct development is arrested. Finally, during the post-diapause phase, the individual emerges from diapause and post-diapause direct development is resumed. All three eco-physiological phases can occur in every stage of the insect life cycle, but an individual species is usually constrained to the diapause phase (developmental arrest) during a single stage of the life-cycle [1]. The diversity of life-cycle timing of eco-physiological phases among insects implies a corresponding diversity of molecular and physiological pathways underlying diapause regulation in different insect species. Most studies on the transcriptional bases of diapause have focused on gene expression during the phase of actual developmental arrest (diapause). However, the pre-diapause phase can reveal important insights into the regulation and physiological trajectory of diapausing animals [19-21].

The Asian tiger mosquito, *Aedes albopictus*, is an outstanding emerging model organism for the study of diapause within a well-defined ecological and evolutionary context. Currently considered the most invasive mosquito species in the world [22], in the last 30 years, *Ae. albopictus *has rapidly spread from its native Asian range across the world and is currently found in at least 28 countries on every continent except Antarctica [22,23]. Temperate populations of *Ae. albopictus *undergo a maternally controlled egg diapause in which exposure of the maternal pupa and adult to short day lengths initiates diapause of the offspring as a pharate larvae inside the chorion of the egg [24,25]. Increased egg desiccation resistance during diapause [20,26] and rapid evolution of both diapause incidence [[27]; Lounibos et al., in press; Urbanski et al., submitted] and diapause timing [Urbanski *et al*., submitted] during the range expansion of *Ae. albopictus *in the US imply that the diapause response has facilitated the rapid global spread of this invasive mosquito. Furthermore, because *Ae. albopictus *is a vector of dengue and Chikungunya viruses, identifying the genetic basis of diapause could potentially provide a platform for developing novel vector control methods [28]. Finally, the complete genome sequence has been determined for *Aedes aegypti *[29], a closely related mosquito in the same subgenus (Stegomyia) as *Ae. albopictus*. The *Ae. aegypti *genome sequence thus provides a valuable "reference genome" that can be used to annotate *Ae. albopictus *transcriptome sequences.

Here, we use a GS-FLX 454 platform to sequence and assemble the transcriptome of *Ae. albopictus *oocytes from females reared under diapause-inducing (DI) and non-diapause-inducing (NDI) photoperiods. Our goals were to 1) generate a transcriptome database for the study of *Ae. albopictus *functional genetics because limited genetic information is currently available for this species; and 2) to identify and verify candidate transcripts involved in the transcriptional bases of diapause preparation. We leverage the close evolutionary relationship of *Ae. albopictus *to *Ae. aegypti *for the annotation of the assembly. We use differential expression, based on normalized read counts from the DI and NDI transcriptomes, to identify a series of candidate genes for diapause preparation, and verify these candidates using quantitative PCR (qPCR). We then discuss the putative functional significance of verified differentially expressed genes relative to the molecular physiology of pre-diapause and diapause in other insects. Our transcriptome data enrich the limited sequence information for *Ae. albopictus*, contribute to our knowledge of gene expression during the pre-diapause phase, and set the stage for comparative analyses both amongst other taxa and relative to other diapause phases of *Ae. albopictus*.

## Results and Discussion

### Sequencing, read cleaning and *de novo *assembly

**Figure 1:**
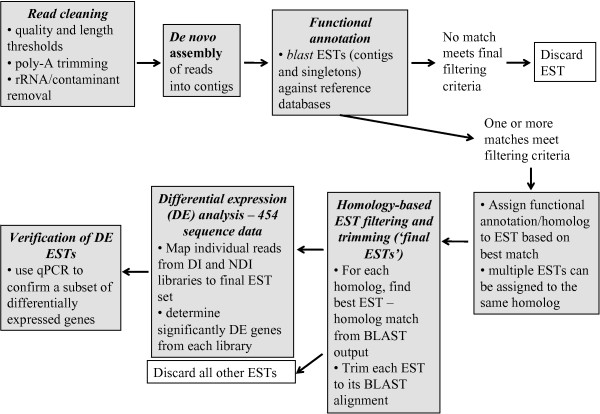
**Flowchart of the main analysis steps outlined in this paper**.

**Figure 2:**
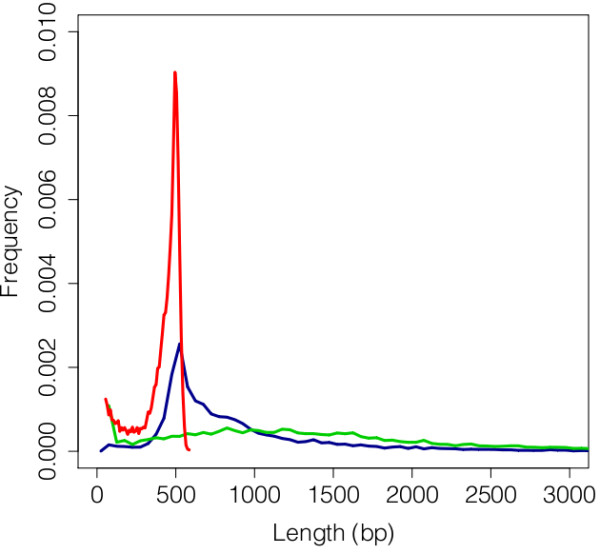
**Distribution of read and contig lengths from *Ae. albopictus *and *Ae. aegypti***. Reads lengths from the *Ae. albopictus *transcriptome are show in red (N = 1,111,941), *Ae. albopictus *contig lengths in blue (N = 69,474), and *Ae. aegypti *transcript lengths (v1.2, http://www.vectorbase.org) in green (N = 18,760).

454 GS-FLX sequencing was performed on cDNA libraries from mature (stage V) oocytes of *Ae. albopictus *females reared under diapause-inducing (DI) or non-diapause-inducing (NDI) photoperiods (see Methods). We merged reads from both libraries for assembly and annotation. Our quality filtering procedure (Figure 1) removed ~14% of the raw sequenced reads, leaving 1,111,941 reads remaining for further analysis. The average read length was 418 bp, with an N50 of 482 bp (Figure 2, Table 1, Table 2). These lengths are broadly comparable with other 454 transcriptome sequencing studies [30-33]. Raw reads and quality scores are archived at NCBI Sequence Read Archive (SRA) under Accession SRP007714.

**Table 1:** Summary statistics for reads from 454 GS-FLX sequencing

	Total # of reads	% reads removed	# of reads remaining after filtering	Average filtered read length	Average filtered %GC	% of reads mapped to contig set	% of mapped, discarded reads
DI	668,269	13.59	570,807	404.11	50.76	61.19	2.62
NDI	656,977	14.91	541,134	432.62	50.75	62.38	3.01
Combined	1,325,246	14.25	1,111,941	417.98	50.76	61.77	2.81

Summary statistics of reads from two cDNA libraries derived from oocytes from DI (diapause inducing) and NDI (non-diapause inducing) photoperiod treatments are shown.

**Table 2:** N50 values, and mean and median lengths, for reads and EST sets

		N50	Mean read length	Median read length
Trimmed reads		482	417.98	467
redundant EST set	Contigs only	1009	898.25	692
redundant EST set	Contigs and singletons	641	601.75	493
trimmed EST set	Contigs only	1120	801.25	647
trimmed EST set	Contigs and singletons	974	688.48	508

Our *de novo *assembly using MIRA 3.2.0 [34] yielded 69,474 contigs, with an N50 of 1,009 bp (Figure 2, Table 2). These contigs are accessible at NCBI''s transcript shotgun archive (TSA) under accession numbers JO845359-JO913491. Contig length is one of the many benchmarks that can be used to assess assembly quality, and our assembly shows a comparable mode of contig length relative to similar studies [30-33]. Annotated transcripts from the *Ae. aegypti *genome, the most suitable reference genome for *Ae. albopictus*, have an N50 of 1,980 bp (Figure 2). This result suggests that many of the contigs in our assembly are shorter than the actual transcripts from which they are derived. This disparity in contig length distributions between *de novo *transcriptome assemblies and annotated transcripts from genome assemblies is expected, because transcript predictions from the genome assembly are based on both extensive computational (automated and manual gene modeling) and empirical (ESTs, cDNA sequences) evidence [29], whereas the *Ae. albopictus *transcriptome is currently limited to data from a single source.

*De novo *assemblies can seldom merge all reads into contigs [35-37], especially when the abundance of some transcripts is too low for representative reads to be assembled. However, the "singleton" reads still represent useful sequence information on low-coverage transcripts, and can be included in subsequent analyses of the transcriptome. In our assembly, 8% of the quality-filtered reads were not assembled [similar to [31,38]], and these singletons were merged with the contig set into a preliminary EST set.

### Functional annotation

**Figure 3:**
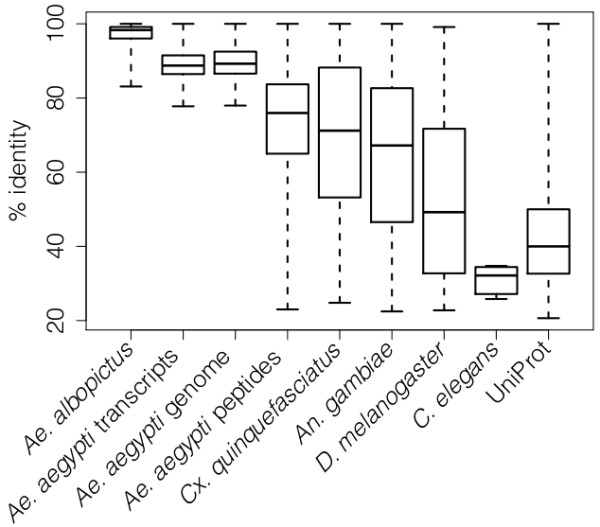
**Box-plot of percent identities from EST BLAST alignments to each reference organism**. The solid horizontal line represents the median, the box encompasses the lower and upper quartiles, and the boxplot whiskers encompasses the data extremes.

To functionally annotate the preliminary EST set, we performed a series of BLAST searches [39] to several reference organisms (Table 3). Our functional annotations drew heavily from two culicid genomes, *Ae. aegypti *and *Culex quinquefasciatus*, due to their close phylogenetic relationship with *Ae. albopictus*. Most ESTs (66%) matched to *Ae. aegypti *transcripts and peptides (Table 4; Figure 3). Percent identities are frequently used as a measure of BLAST quality [35,40]. Here, percent identities between the ESTs and their putative homologs declined with phylogenetic relatedness, with the highest percent identities found to putative homologs in *Aedes spp.*, and the lowest to *C. elegans *(Figure 3). 28% of ESTs (4% contigs, 24% singletons) had no significant similarity to any of the databases that we searched, likely due to the conservative e-value cutoffs we used when assigning homology (Table 3).

**Table 3:** Databases used for BLAST homology searches with relevant details for each search

Reference organism	Sequence type	BLAST algorithm	Minimum e-value	Version	Source	Minimum % identity used in final EST set selection
*Ae. albopictus*	mRNA	BLASTN	1.00E-10	N/A	http://www.ncbi.nlm.nih.gov	85
*Ae. aegypti*	transcripts	BLASTN	1.00E-10	AaegL1.2	http://www.vectorbase.org	85
*Ae. aegypti*	genomic	BLASTN	1.00E-10	AaegL1	http://www.vectorbase.org	85
*Ae. aegypti*	peptides	BLASTX	1.00E-10	AaegL1.2	http://www.vectorbase.org	70
*Cx. quinquefasciatus*	peptides	BLASTX	1.00E-05	CpipJ1.2	http://www.vectorbase.org	0
*An. gambiae*	peptides	BLASTX	1.00E-05	AgamP3.6	http://www.vectorbase.org	0
*D. melanogaster*	translation	BLASTX	1.00E-05	r5.29	http://www.flybase.org	0
*Ca. elegans*	peptides	BLASTX	1.00E-04	WS218	http://www.wormbase.org	0
Swiss-Prot	proteins	BLASTX	1.00E-03	2010_6	http://www.uniprot.org	0

The minimum e-value used for the initial EST set, and the minimum % identity used for the final EST set, varied based on phylogenetic distance of the organism/database and *Ae. albopictus*.

**Table 4:** Number of ESTs assigned to each genomic database

Reference organism	Number of matches, initial EST set	Number of matches, final EST set
*Ae. albopictus*	1,011	52
*Ae. aegypti *transcripts	91,461	10,005
*Ae. aegypti *genome, annotated	1,422	142
*Ae. aegypti *genome, unannotated	10,736	N/A
*Ae. aegypti *peptides	2,993	556
*Cx. quinquefasciatus*.	1,167	550
*An. gambiae*	103	79
*D. melanogaster*	21	16
*C. elegans*	25	19
Swiss-Prot	4,331	86
total number of matches	113,270	11,505

no hit (contigs)	6,775	N/A
no hit (singletons)	37,077	N/A
total number of contigs	69,474	N/A
total number of singletons	87,648	N/A

Both the preliminary EST set and final, trimmed EST set are listed (see Methods).

### Homology-based EST filtering and trimming

The MIRA assembler tends to assemble contigs with high redundancy [32,35,40], and our dataset is no exception: on average, more than eight ESTs matched the same gene (min = 1; median = 4; mean = 8.86; max = 5,613; See additional file 1: Distribution of the number of ESTs assigned to a reference gene). There are both biological and technical reasons for this high redundancy. For example, alternatively spliced isoforms, or alleles of the same gene, could assemble into separate contigs. Additionally, sequencing errors, incomplete transcript coverage during sequencing, chimeric reads or contigs, or paralogous genes from recently diverged gene families could also contribute to the redundancy. Biologically valid transcript variants are difficult to distinguish from sequencing and assembly errors, particularly in the absence of a reference genome. To systematically reduce our EST set to a non-redundant transcriptome, we took advantage of the homology of the *Ae. albopictus *ESTs to the closely related *Ae. aegypti *genome, as well as homology to other, more distantly related genomes. For each group of ESTs that matched a putative homolog, we identified one ''representative'' EST, and trimmed this EST to its overlap with the putative homolog (see Methods). This resulted in 11,561 non-redundant, ''conservative'' ESTs with annotations to known genes. In the subsequent text, we refer to this non-redundant, trimmed EST set as the ''final'' EST set. The N50 of the final EST set improved over that of the redundant ESTs (Table 2). While this conservative approach certainly removed perfectly legitimate sequence data, it systematically avoided chimeric contigs that could occur due to contig mis-assembly, thereby yielding a higher-confidence EST set. The advantage of this approach is that it eliminated redundant ESTs, as well as annotations with little support from other organisms. The disadvantages are that genes novel to *Ae. albopictus *are eliminated, and that some valid sequence information from ESTs with homology information was discarded. A similar approach was used by Crawford *et al*. [41] for a non-model mosquito species (*Anopheles funestus*). Here, the authors utilized significant homology of their ESTs to other reference genomes to validate contigs and eliminate redundancy, reflecting the fact that EST redundancy and uncertainty are common problems in transcriptome assembly of organisms without a genome sequence, and that unique, taxon-specific approaches will often be necessary.

Trimmed ESTs covered an average of 43% of their putative homologs (See additional file 2: Box plot of the percent length of each reference gene matched by its *Ae. albopictus *EST putative homolog), which indicates that the majority of the final ESTs do not represent full transcripts. This result is expected, given the incomplete nature of *de novo *transcriptome assembly of non-model organisms, as well as our rigorous trimming procedure. Other *de novo *transcriptome assemblies of mosquito species have also documented incomplete transcript coverage based on comparisons to a closely related reference genome [41,42]. Of the ESTs that remained in the final contig set, 93% were annotated to *Aedes *spp. (Table 4), with high percent identities (mean: 86.4%; See additional file 3: Box-plot of percent identities from BLAST alignments of final ESTs to each reference organism). Not surprisingly, percent identities of BLAST matches to other, less closely related organisms were much lower (mean: 59.4%, See additional file 3: Box-plot of percent identities from BLAST alignments of final ESTs to each reference organism). We provide fasta files of the trimmed ESTs, as well as an Excel spreadsheet with relevant annotation information at http://AlbopictusExpression.org.

To evaluate whether our sequencing efforts maximized the number of transcripts captured, we generated a bootstrapped gene accumulation curve [31], based on BLASTN matches of individual reads to the *Ae. aegypti *transcriptome. After an initial steep increase in the number of *Ae. aegypti *transcripts discovered with increasing read number, the slope of the curve rapidly asymptotes, which implies that our sequencing depth captured most of the *Ae. aegypti *transcript homologs found in our cDNA libraries (See additional file 4: Gene accumulation curve of *Ae. albopictus *ESTs). Paired with other results indicating that our coverage of putative homologs is incomplete (See additional file 2: Box plot of the percent length of each reference gene matched by its *Ae. albopictus *EST putative homolog), we conclude that our assembly maximized the number of putative homologs found in our libraries, but that the ESTs representing these homologs are incomplete. Because of this, our measures of expression under DI and NDI conditions will underestimate actual expression, although we do not expect a bias in this estimate between DI and NDI libraries. However, differentially expressed genes with inherently low expression are more likely to go undetected than genes with high expression, in particular when sequencing coverage is low [43].

### Differential expression (DE) analysis- 454 sequence data

**Figure 4:**
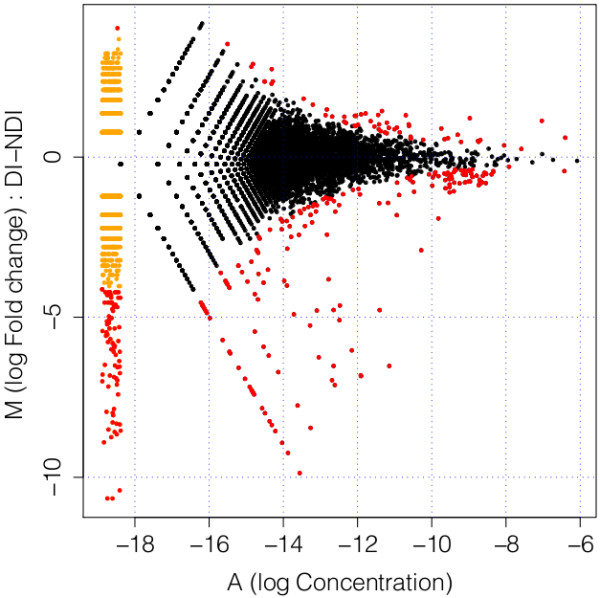
**Log fold-change expression (M) versus log abundance (A) of TMM-normalized expression from the 454 dataset**. The log fold-change and log abundance for genes that are unique to a treatment is undefined; therefore, these genes are shown at an arbitrarily low abundance on the left of the plot in orange. Genes with higher expression under diapausing (DI) conditions have positive M values, and genes with higher expression under non-diapausing (NDI) conditions have negative M values. Genes that qualified as significantly differentially expressed (corrected p < 0.001) are in red.

We estimated expression levels for each EST under DI and NDI treatments by mapping reads from each treatment to the final EST set. 62% and 61% of reads from the NDI and DI libraries, respectively, uniquely mapped to the final EST set (Table 1). 2.8% of all reads were discarded because they did not map uniquely. Almost all remaining unmapped reads corresponded to the untrimmed EST set (data not shown); those that did not had low complexity regions over the majority of the read length, and thus were not assembled. Some viral and bacterial genes (55) were discovered during the annotation process. These genes were retained for read mapping, but the genes were removed before measuring differential expression, as measuring viral loads was outside the scope of this study. Using TMM-normalized read counts [Trimmed Mean of M values, [44]] to initially determine differential expression of ESTs between photoperiod treatments (see Methods), 257 ESTs had significantly higher expression under NDI conditions, and 57 ESTs were over-expressed under DI conditions (Figure 4; See additional file 5: Characteristics of all differentially expressed genes from the 454 dataset). Additionally, the range of log fold-change tended to be higher under NDI relative to DI conditions (Figure 4).

### Verification of DE ESTs by qPCR

**Figure 5:**
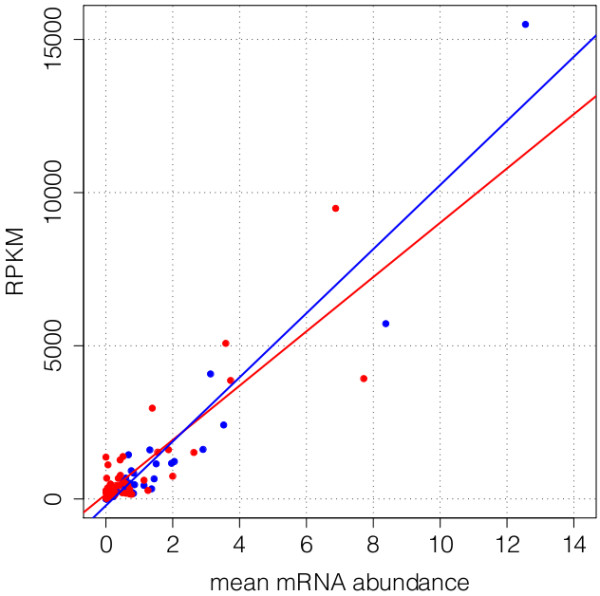
**RPKM values (454 sequence data) versus mean qPCR mRNA abundance for NDI and DI treatments**. NDI treatments are shown in red, DI in blue. Lines from the linear regressions for each photoperiod treatment, after removal of the outlier histone2A, are plotted as solid lines (r^2^(NDI)= 0.70; r^2^(DI)= 0.90).

We evaluated differential expression of 48 candidate genes using qPCR to validate expression patterns in the 454 dataset. Of these, 21 were predicted over-expressed under NDI conditions, 10 under DI conditions, and 17 were of *a priori *interest. We first asked whether expression levels from the 454 dataset and qPCR experiments were correlated, and whether the normalization method of the 454 dataset affected these correlations. In addition to the TMM method that we used to identify candidate genes [44], we calculated RPKM (Reads Per Kilobase per Million, [45]), and asked whether these two measures correlated with mean mRNA abundance from the qPCR experiments for NDI and DI treatments. We removed one gene with much higher qPCR than 454 expression because it is extremely likely that paralogs were co-amplified by qPCR but were undetected by the qPCR melt-curve analysis. This gene, a histone 2A homolog (*Ae. aegypti *ID AAEL000494), has 19 near-identical paralogs in *Ae. aegypti*, of which eight are 100% identical at the peptide level (http://www.vectorbase.org). Subsequently, correlation coefficients of differential expression for the remaining 47 candidate genes assessed by 454 sequencing and qPCR were high and resembled those found in previous comparisons of qPCR and RNA-Seq data (Figure 5; Table 5; comparisons in [46]: r^2 ^= 0.711-0.807). Correspondence between 454 and qPCR expression levels was notably higher when 454 expression was measured as RPKM, suggesting that RPKM is a more accurate measure of gene expression for our dataset.

**Table 5:** Correlation coefficients (r^2^) between qPCR mRNA abundance and 454 gene expression

normalization method	NDI	DI
RPKM	0.722	0.911
TMM	0.548	0.727

Correlation coefficients are derived from linear regressions between qPCR mRNA abundance and both TMM and RPKM gene expression measures from 454 read counts for each gene verified by qPCR.

While correlation coefficients between 454 expression and mRNA abundance calculated within each photoperiod treatment were very high for expression measures in the DI treatment, correlation coefficients were lower for expression measures from the NDI treatment for all analyses (Figure 5; Table 5). This raises the question whether this difference is biological (genes are inherently more variable under longer photoperiods) or technical (differences in library or material preparation, as well as stochastic differences, could have resulted in lower correlations in the NDI dataset). To address this question, we calculated coefficients of variation (CVs) of mRNA abundance from the five replicates of the qPCR data for each gene and photoperiod treatment. If gene expression were more variable in animals reared under NDI conditions, we would expect higher CVs of mRNA abundance for NDI vs. DI treatments. While the median CV was marginally higher among NDI replicates (0.187 vs. 0.173), CVs were not significantly different between photoperiod treatments (paired Student''s t-test, p = 0.38). This result suggests that the lower correlation between qPCR and 454 data in the NDI dataset is more likely due to technical artifacts, rather than inherently higher variation in gene expression under NDI conditions.

### Verification of DE ESTs - Significance comparisons

Perfect congruence between the levels of significance detected using qPCR and 454 sequencing is not to be expected, because 1) the 454 results were based only on one biological replicate, thus increasing the possibility of both type I and type II error, and 2) the datasets were acquired using fundamentally different approaches, necessitating alternative analyses to determine significance of DE. While qPCR has some methodological caveats [47], it is generally considered a benchmark for gene expression analyses. To determine whether significant differential expression in the 454 dataset corresponds to significant DE in the qPCR data, we calculated sensitivity (true positive rate) and specificity (true negative rate) statistics of the 454 dataset. Because our correlation analyses between 454 and qPCR expression levels suggest that RPKM performs better at predicting mRNA abundance than the TMM normalization, we compared both expression measures'' performance for predicting qPCR significance. Despite the better predictive ability of RPKM on mRNA abundance, both expression measures of the 454 data were poor predictors of qPCR significance (Table 6). This is perhaps not surprising, given the dramatically different methods used to determine significance between 454 and qPCR noted above. Despite lack of congruence between 454 and qPCR results regarding genes predicted as significantly DE, 10 of 10 genes from the DI category had higher average expression under DI conditions based on qPCR results, and as such were directionally consistent between both methods (See additional file 6: Results of qPCR validation). As expected based on the lower correlations in the NDI data noted above, directional consistency was lower for NDI genes (9 of 21). These results indicate that 454 sequencing as applied in this study can be useful guide to *de novo *identification of transcriptional differences underlying complex phenotypes, but that replication, either by verification with qPCR, or by sequencing replicated libraries, is essential for verification. Below, we restrict our discussion of transcriptional elements of diapause response in *Ae. albopictus *to ESTs verified as DE by qPCR.

**Table 6:** Sensitivity and specificity information calculated from 48 candidate genes

	Normalization method	
	RPKM	TMM
TP (true positives)	4	6
TN (true negatives)	19	12
FP (false positives)	19	26
FN (false negatives)	5	3
TPR (sensitivity)	0.44	0.67
TNR (specificity)	0.50	0.32

Values for both RPKM and TMM expression measures from the 454 data are shown. Sensitivity is calculated as the number of true positives over the number of all positives; Specificity is the number of true negatives divided by all negatives.

### Verification of DE ESTs - Functional categories of verified genes

Several themes in the insect diapause program have emerged from comparative analyses of diapause-mediated developmental arrest, including DNA replication and transcription, endocrine signaling, metabolism, and response to stress [3-5,8,9,21]. These processes can be important during diapause preparation, but other pathways leading to preparatory functions, such as growth regulation in advance of developmental arrest, accumulation of metabolic reserves, and behavioral or morphological changes to protect against the physiological stresses of the harsh environment [1], may dominate the transcriptional profile during the preparatory phase. In *Ae. albopictus*, maternally provisioned transcripts from females reared under short day lengths dictate the development of diapause-destined oocytes; it is not known in *Ae. albopictus*, nor in most other pre-diapause insects, which of these processes should be transcriptionally dominant. From qPCR experiments, we found differential expression of ESTs belonging to the main categories of developmental arrest during diapause, and identified additional ESTs from pathways that have not yet received attention in previous studies on the molecular bases of diapause (Table 7).

**Table 7:** Genes verified as DE from qPCR analyses

*Ae. albopictus *ID	Putative homolog	454 category	Fold-change	corrected p-value	Functional category
Aalb_oocyte_rep_c42113_trimmed	phosphoenolpyruvate carboxykinase (*pepck*)	DI	0.82	0.004	metabolism
Aalb_oocyte_rep_c2808_trimmed	Ecdysone inducible protein L2, putative (*eip*)	NDI	-0.85	0.005	endocrine signaling
Aalb_oocyte_rep_c41764_trimmed	Inhibitor of growth protein (*ing1*)	*a priori*	1.05	0.002	DNA replication/ transcription
Aalb_oocyte_rep_c38864_trimmed	Conserved hypothetical protein (AAEL004873)	NDI	0.48	0.004	unknown
Aalb_oocyte_rep_c36433_trimmed	Conserved hypothetical protein (AAEL008645)	DI	1.33	0.000	unknown
Aalb_oocyte_GH79BIP01BENVH_trimmed	Bhlhzip transcription factor bigmax	DI	0.87	0.010	DNA replication/ transcription
Aalb_oocyte_rep_c3449_trimmed	GPCR Methuselah Family	*a priori*	0.88	0.004	stress response
Aalb_oocyte_rep_c40438_trimmed	Receptor for activated C kinase, putative (*rack1*)	DI	0.86	0.003	endocrine signaling
Aalb_oocyte_rep_c18194_trimmed	Conserved hypothetical protein (AAEL012019)	DI	0.92	0.010	unknown
Aalb_oocyte_rep_c431_trimmed	phosphatidylethanolamine-binding protein (*pebp*)	*a priori*	0.88	0.000	morphogenesis

Information on each gene verified as differentially expressed is shown. Putative homologs were derived from BLAST searches (see Methods). "454 category" refers to the differential expression status assigned to the EST based on read counts: DI or NDI, significantly over-expressed under DI or NDI conditions, respectively; and *a priori*, not DE based on 454 read counts, but of *a priori *interest based on previous diapause research. Fold-change calculations are based on qPCR results, and were calculated as log2(DI-NDI), where DI and NDI are the average mRNA abundance for DI and NDI treatments, respectively (see Methods). The corrected p-value is derived from Student''s t-test of mRNA abundances derived from qPCR reactions.

Developmental arrest is the definitive diapause phenotype. Studies of pre-diapause gene expression in other insects suggest that precursors of developmental arrest are initiated during the preparatory phase [17,21]. In our study, two ESTs with homologs involved in DNA replication and transcription were more abundant in DI oocytes: *inhibitor of growth protein *(*ing1*, AAEL003650) and *bhlhzip transcription factor bigmax *(AAEL011202) (Table 7). In various systems, ING1 has shown involvement in oncogenesis, apoptosis, DNA repair and negative cell cycle regulation [reviewed in [48]]. In *D. melanogaster*, ING1 is thought to interact with p53, which is a transcription factor that can respond to stress by affecting cell-cycle arrest, DNA repair, apoptosis, or senescence [49]. Cell-cycle arrest is one of the unifying themes of diapause [9], thus genes that may affect this arrest are good candidates as developmental regulators. Among its various functions, the transcription factor *Bhlhzip bigmax *appears to be a target of FOXO and is involved in regulating metabolism and energy sensing [50,51]. The importance of FOXO in the diapause of *Culex pipiens*, another mosquito [52], and the critical role of energy sensing during diapause [8] suggests possible roles for these two ESTs in the preparatory phase of diapause in *Ae. albopictus*.

The endocrine system plays a central role in regulating diapause [53]. Although our understanding of the endocrine regulation of embryonic diapause is restricted to just a few species, high levels of ecdysteroids are critical for the induction and maintenance of embryonic diapause in the gypsy moth, *Lymantria dispar *[54]. In pre-diapause *Ae. albopictus *oocytes, two homologs of genes potentially involved in ecdysone signaling were differentially expressed. The first, *rack1*, encodes a receptor for activated protein kinase C, and was more abundant under DI conditions. RACK1 is known to bind to several different signaling molecules [55], including a molt-associated transcription factor that is linked to the action of 20-hydroxyecdysone [56]. It is highly expressed in ovary tissue and is thought to be required for oogenesis in *D. melanogaster*; female *D. melanogaster *homozygous for RACK1 null alleles show reduced ovary size [55]. *rack1 *has also been implicated in diapause in other insects. For example, in a study of gene expression in pre-diapause and diapause cricket embryos (*Allonemobius socius*), *rack1 *expression was higher in pre-diapause embryos, but subsequently decreased during diapause [21]. Intriguingly, it has recently been shown that RACK1 affects circadian rhythm function in mouse [57]. A classic hypothesis proposes that circadian rhythm underlies the expression of photoperiodic diapause [58], although evidence supporting this hypothesis is limited [59,60] and highly debated [e.g. [61]]. However, these findings suggest a possibly complex role for RACK1 in diapause preparation. The second differentially expressed transcript potentially related to endocrine signaling is ecdysone inducible protein L2 (*eip*), which was less abundant in DI oocytes. The *D. melanogaster *homolog of eip, *imp-l2*, has been implicated in ectoderm and neural development [62], is essential for starvation resistance [63], and may have a role in the regulation of growth [64]. Loss-of-function alleles of *imp-l2 *in *D. melanogaster *result in female size increase, which appears to be primarily driven by enlarged ovaries [63]. Interestingly, both *rack1 *and *eip *have phenotypes related to ovary size in *D. melanogaster *[55,63]. Eggs from *Ae. albopictus *reared under DI conditions are larger and contain more lipids than eggs from females reared under NDI conditions (PAA, unpublished data); we speculate that decreased pre-diapause expression of the *Ae. albopictus imp-l2 *homolog, and/or increased expression of the *rack1 *homolog, could affect ovary and subsequent egg size. Ongoing studies of the expression levels of these genes during embryological development and developmental arrest in pharate *Ae. albopictus *larvae will provide further insight into the diapause-related function of these transcripts, as well as important comparative results regarding the ecdysone signaling pathway.

Diapause is often accompanied by a decrease in metabolism. One gene involved in metabolism was more abundant in DI oocytes: phosphoenolpyruvate carboxykinase (*pepck*). PEPCK is part of the gluconeogenesis pathway, and has higher expression levels during diapause in *Sarcophaga crassipalpis *[5]. Diapausing insects appear to enhance gluconeogenic pathways, in part due to a shift towards anaerobic metabolism [8]. We hypothesize that the over-expression of this transcript during the pre-diapause phase in *Ae. albopictus *oocytes may either represent a maternally provisioned regulatory cue, or initiation of the gluconeogenic pathway in advance of the onset of developmental arrest. The transcription factor *Bhlhzip bigmax*, discussed above, could also contribute to the down-regulation of metabolism [50,51] associated with diapause.

Increased longevity and stress resistance are also important features of the diapause program. Potentially relevant to these phenotypes, a gene encoding a G-protein coupled receptor (GPCR) from the *Methuselah *family was more abundant in pre-diapause oocytes. In *D. melanogaster*, the *methuselah *(*mth*) gene is associated with longevity and stress resistance [65]. *Mth *haplotype distributions vary latitudinally in *D. melanogaster *[66], and studies of allelic variation in *mth *suggest that the gene contributes to differences in lifespan among populations [67]. While down-regulation of *mth*, rather than over-expression, should result in greater stress resistance based on results from *D. melanogaster*, other GPCRs are potentially up-regulated in response to diapause-related stimuli in other organisms. Some GPCRs in *C. elegans *show strong responses to *dauer *formation [68], which is analogous to insect diapause. In *Bombyx*, a GPCR shows high affinity to diapause hormone [69], suggesting it could be important in mediating developmental arrest.

Three other genes that did not fall into established functional categories related to diapause had higher expression in pre-diapause oocytes. The putative *Ae. aegypti *homologs of all three genes are annotated as "conserved hypothetical proteins". These genes may be particularly intriguing, as they could relate specifically to diapause preparation in *Ae. albopictus *or other insects. However, because their annotations are uncertain, the genes'' functions are highly speculative. We provide brief descriptions of the genes'' domains and gene ontology categories, if available, recognizing that much more work is necessary to understand the function of these genes in the context of *Ae. albopictus *diapause. AAEL008645''s gene ontology association is ''protein binding''. It contains evidence for a B30.2/SPRY domain, a LisH motif, and a CRA domain. AAEL004873''s gene ontology associations are protein binding and cell adhesion. It contains a GILT motif (gamma-interferon-inducible lysosomal thiol reductase), which is thought to be associated with disulphide bond reduction. While it was predicted to be over-expressed in non-diapause destined oocytes based on RPKM, qPCR experiments show that it is actually more abundant in pre-diapause oocytes. AAEL012019 has several motifs; a galactose-binding-domain-like motif (sub-motif: coagulation factor 5/8 type, c-terminal; Muskelin, n-terminal; Kelch1); and a LisH dimerization motif.

Previously, we investigated the expression patterns of a putative phosphatidylethanolamine-binding protein (*pebp*) in diapause and non-diapause oocytes in multiple populations of *Ae. albopictus *[19]. While our present data show significant up-regulation of *pebp *in a single temperate population under DI conditions, the previous study suggested a regional effect on *pebp *expression in these populations, rather than a simple over-expression of *pebp *under DI conditions. These results are not necessarily contradictory, as the studies differ in their experimental design and replication: while the previous study utilized more populations, our analysis includes more replicates per photoperiod treatment, and thus may have more statistical power to capture subtle differences in expression levels. This could indicate that the differential expression of *pebp *under different photoperiods is population-specific. There is little information about the role of *pebp *in insects, but studies from plants have documented differential expression of a *pebp *gene family member FT (Flowering locus T) in response to photoperiod [70], which shows differential expression to photoperiod treatments in latitudinally disparate populations in *Picea abies *[71]. These results raise the intriguing possibility that genes from the *pebp *family may have similar roles or be involved in photoperiodically mediated life history transitions across both plant and animal kingdoms.

Three additional genes (*fatty acyl coA elongase*, gi|239997749|; *heat shock protein 67B2*, gi|254728755|; and *epithelial membrane protein*, gi|270037306|) implicated in the diapause response of *Ae. albopictus *based on differential abundance in oocyte tissue [19,20] exhibited non-significant up-regulation under DI conditions in the current study. Because the experimental design of the previous studies differed from the current experiments, exact comparison of results is not possible. Nevertheless, the direction of differential expression is the same, indicating that our current results are qualitatively consistent with these previous studies.

## Conclusions

Our goals for this study were: 1) to generate a comprehensive oocyte transcriptome for *Ae. albopictus*, an emerging model system for studying the evolutionary and ecological genomics of diapause, and 2) to identify candidate genes for diapause preparation in this species. Our assembly of a high-quality, conservative transcriptome for *Ae. albopictus *oocytes highlights useful approaches to utilizing a closely related reference genome to generating high-confidence ESTs in a non-model organism. Additionally, the assembly enabled us to identify several candidate genes for diapause preparation that are consistent with established themes of the insect diapause program as well as additional candidates that are potentially unique to *Ae. albopictus*. In addition to the potential relevance to diapause, many of these ESTs are related to fundamental biological processes such as metabolism, stress tolerance, and the endocrine control of development and thus may ultimately provide useful targets for developing novel forms of vector control in this medically significant vector of human disease. Finally, the comprehensive oocyte EST database should provide a useful resource for comparative genomics and vector molecular physiology.

## Methods

### Tissue generation for 454 sequencing from *Ae. albopictus *oocytes

We collected over 400 *Ae. albopictus *larvae and pupae from approximately 20 tires located at a used tire yard in Manassas, VA, in 2008. This strain was reared in the laboratory on a non-diapause inducing (NDI) long-day photoperiod (16 h light, 8 h dark) at 21°C and ca. 80% relative humidity for five generations as described in Armbruster and Hutchinson [72] and Armbruster and Conn [73]. To produce tissue for transcriptome sequencing, in the laboratory F_6 _generation approximately 200 female pupae were placed into each of two cages, one of which was maintained under an NDI photoperiod (16 h light, 8 h dark) and the other of which was maintained under a diapause-inducing (DI) unambiguous short-day photoperiod (8 h light, 16 h dark). Both cages were maintained at 21°C and ca. 80% relative humidity. Females were bloodfed to repletion 7-18 days after eclosion on a human host. Although we included females from an 11-day range of chronological age, this variation is unlikely to have a large effect on the abundance of mature oocyte transcripts since ovarian development is more strongly influenced by time since blood meal than chronological age [74]. Five days after bloodfeeding, females were anaesthetized with CO_2 _and frozen at -80°C. Mature (stage V) oocytes were identified based on a visible exochorion pattern and dissected into RNAlater^TM ^(Sigma Aldrich, St. Louis, MO). 60 - 80 frozen mosquitoes per photoperiod treatment were used.

### Tissue generation for qPCR

Tissue for qPCR reactions was generated for 5 biological replicates under each of NDI and DI photoperiod treatments using the same methods described above, but from F_7 _(2 replicates) and F_8 _(3 replicates) laboratory generations. Briefly, ca. 50 female pupae for each biological replicate were placed in separate adult cages under NDI and DI photoperiod treatments as described above. Oocytes from 12 to 27 females were used for each biological replicate from each photoperiod treatment.

### Diapause incidence measurements

We confirmed the diapause response for each generation and replicate of laboratory rearing. Adult cages were established under DI and NDI photoperiod treatments as described above with ca. 50 male and 50 female mosquitoes per cage. Females were blood fed to repletion on a human host and a small black jar half-filled with ca. 20 ml of dI water and lined with an unbleached paper towel was placed into each cage. We collected the towels with oviposited eggs every Monday, Wednesday, and Friday. Two days after collection the towels were slowly dried and stored at ca. 80% relative humidity. The diapause response was measured by stimulating 10-20 day old eggs to hatch following Novak and Shroyer [75]. Egg towels were re-dried and hatched a second and third time after 1 and 2 weeks, respectively, to ensure hatching of all non-diapausing eggs. Embryonated, unhatched pharate larvae (in diapause) were identified by submerging eggs in a bleach solution for 48-72 h [76] and percent diapause incidence was calculated as the number of embryonated but unhatched larvae divided by the total number of viable pharate larvae in the sample (embryonated unhatched and hatched larvae) [27,77].

### RNA preparation and sequencing

We extracted total RNA using TRI^® ^Reagent (Sigma Aldrich, St. Louis, MO) followed by an isopropanol precipitation, according to manufacturer''s instructions. DNA was removed from each sample with Turbo-DNAfree (Applied Biosystems/Ambion, Austin, TX) and RNA integrity assessment was performed for each sample on an RNA chip (Bioanalyzer 2100, Agilent Technologies, Santa Clara, CA). For RNA used in 454 sequencing, we enriched for mRNA with Dynabeads oligo(dT) probes (Dynal Biotech, Oslo, Norway). mRNA was then sent to the University of Maryland Institute for Genome Sciences for 454 GS-FLX Titanium sequencing using standard protocols (Roche, Inc.). Briefly, mRNA was fragmented with a zinc chloride solution. cDNA was synthesized from the fragmented mRNA using random hexamer primers, and separate adaptors were ligated to cDNA fragments from each cDNA library (DI and NDI). Fragments were then sequenced with GS FLX chemistry. Raw reads and quality scores are archived at NCBI Sequence Read Archive (SRA) under Accession SRP007714; trimmed EST sequences are accessible at NCBI''s transcript shotgun archive (TSA) under accession numbers JO845359-JO913491. We also provide the raw reads, trimmed ESTs, untrimmed contigs, and an Excel spreadsheet with relevant annotation information at the **Aedes albopictus expression database **[http://AlbopictusExpression.org].

### *De novo *transcriptome assembly and annotation - read cleaning

A flow chart describing our data analysis workflow is presented in Figure 1. Reads were trimmed according to pre-defined Roche settings prior to assembly. As additional quality filters, we eliminated 1) reads that contained at least one ambiguous base [78]; 2) duplicate reads, which are known to occur as an artifact of 454 sequencing [79]; 3) reads with an average quality score lower than 25 [following 78, as well as our own observations of read quality]; and 4) reads with significant BLAST matches (e-value > 1e^-25^) to *Ae. albopictus *rRNA and to *Wolbachia *(See additional file 7: Gene IDs of *Ae. albopictus *rRNA and *Wolbachia *spp. used to pre-screen reads). We used the program SnoWhite 1.1.4 [[80], unpublished] to trim poly-A/T tails and to remove all reads shorter than 50 bp, and ssaha2 [81] to match adaptor sequences to reads, which were then masked in the assembly program MIRA [34].

### *De novo *transcriptome assembly and annotation - *De novo *assembly

We used MIRA 3.2.0 [34] to assemble pooled reads from both libraries into a single contig set. Default assembly parameters for ESTs generated by 454 sequencing technology were used with minor modifications to permit the assembly of contigs with SNPs (See additional file 8: MIRA command line used for transcriptome assembly). These modifications were required because we sequenced an outbred population of laboratory-reared mosquitoes, rather than an inbred line. MIRA has recently been shown to be one of the more reliable programs for *de novo *454 transcriptome assembly [35]. To assess whether the amount of sequence used affected contig length, we performed a test assembly in MIRA with one half of the 454 dataset. Additionally, we performed exploratory assemblies with Newbler 2.3 [82], which yielded qualitatively similar results (data available on request from MFP). However, because more gene models were captured with the MIRA assembly, as assessed by BLAST matches, we used the MIRA assembly for further analyses.

### *De novo *transcriptome assembly and annotation - functional annotation

We performed a series of BLAST searches to establish homology between contigs or singletons (both referred to hereafter as "ESTs") from *Ae. albopictus *and annotated genes from other organisms. Because of the limited EST information available on NCBI for *Ae. albopictus*, we relied mainly on annotations and sequence information from *Ae. aegypti*, a closely related mosquito in the same sub-genus (*Stegomyia)*. We performed BLASTN and BLASTX searches of our EST dataset to several reference databases with increasing e-value cutoffs as taxonomic distance increased (Table 3). The match with the lowest e-value was retained. If multiple matches with the same e-value were present, then the match with the highest bitscore was retained. Our search against *C. elegans *proteins was inspired by Ragland *et al*. [5], who found limited evolutionary conservation in dormancy expression patterns between *Sarcophaga crassipalpis *and the well characterized larval dauer stage of *C. elegans *[68].

To evaluate whether we had recovered close to the maximum number of genes contained in our sequencing libraries, or whether greater sequencing depth of these libraries would have led the recovery of more unique genes, we generated a bootstrapped gene accumulation curve [e.g. [31]]. We used BLASTN to determine homology between individual reads and *Ae. aegypti *transcripts, using a minimum required e-value of 1e^-10^. We randomized the BLAST output order 1,000 times, and then calculated the mean number of *Ae. aegypti *transcripts captured by each additional read. We then plotted the mean cumulative number of new *Ae. aegypti *transcripts discovered with each new read; if sequencing depth was sufficient, then this curve should reach an asymptote with an increasing number of sampled reads.

### *De novo *transcriptome assembly and annotation - homology-based EST filtering and trimming

Our BLAST searches revealed high redundancy in the ESTs, such that multiple ESTs had the closest similarity to the same putative homolog, and often to the same region of that homolog (mean/median number of ESTs per gene: 8.6/4; See additional file 1: Distribution of the number of ESTs assigned to a reference gene). This redundancy is often observed with the MIRA program [83], and could reflect biological variation among transcripts originating from a single gene, for example alternative splicing or allelic variation. However, this result could also be due to sequencing errors, or spuriously assembled sequence. Many of the redundant contigs assigned to a reference gene were highly diverged in their 5'' and 3'' ends. To determine whether these variable ends represent alternative isoforms of a gene or are due to sequencing or assembly errors, we amplified these alternative ends using qPCR. This confirmed the presence of some alternative isoforms (amplified product size was consistent with predicted product size from contigs in 18 out of 22 cases, data not shown). However, due to the high frequency of redundant ESTs, which makes it impossible to confirm all isoforms comprehensively, we opted take a conservative approach and only use the region of one, "representative" EST that aligned with its homolog for further analyses (Figure 1). This method will remove redundancy and uncertain annotations, but will also remove valid sequence (see below), and sequences unique to *Ae. albopictus*.

To identify representative ESTs, for each group of ESTs assigned to the same gene, we found the EST with the best tradeoff between length and % identity. To do this, we selected ESTs with a % identity above a set value (Table 3; % identity cutoffs were chosen based on visual inspection of the distribution of % identity values from matches to each genome database). Within this subset, we chose the EST with the longest BLAST match. When the % identity of all ESTs assigned to a gene was lower than the cutoff, the match with the highest % identity was used. For EST groups with matches in all other databases, the match with the highest % identity was used (Table 3), as % identity was generally lower in these groups; length was not included as a selection criterion, other than a minimum length requirement of 50 bp. Matches to the *Ae. aegypti *genome were only used in further analyses if the majority (>50%) of the EST was contained within an annotated mRNA (gff3 AaegL1.2). Once the best match for a reference gene was determined, we trimmed the aligned EST to its start and stop coordinates from the BLAST output, to eliminate variable 5'' and 3'' ends that could represent either alternative isoforms, or sequencing and assembly error. On average, 464 bp were trimmed from each EST (min: 1bp, max: 10,810 bp).

### Differential expression (DE) analysis - 454 sequence data

To identify candidate DE genes from our 454 dataset, we estimated expression for each EST based on counts of mapped reads to the trimmed ESTs. We mapped reads from each cDNA library (DI and NDI) to the filtered and trimmed EST set using the program ssaha2 [81] with 454 default settings, with the additional requirement of a minimum of 95% identity. Reads that mapped to multiple ESTs or locations (2.8%) were discarded.

We used two different measures of differential expression (DE). First, we performed the TMM normalization method (trimmed-mean of M values) as implemented in the *edgeR *package [[44], http://www.bioconductor.org/]. This method calculates a normalization constant to account for differences in library size. Additionally, it corrects for biases in read count data that arise when the total number of expressed genes are skewed towards one library, a phenomenon that we had observed in our read counts. We used this normalization method to guide our choice of candidate genes for qPCR validation (see below).

Factors in addition to library size and skew can influence read counts in RNA-Seq data. For example, transcript length is also known to affect read abundance [45,84]. As a second evaluation of expression, we calculated RPKM [the number of reads per kilobase of exon model per million mapped reads, [45]]. We used trimmed EST length as transcript length.

To calculate the significance of differences in normalized read counts, we used the sage.test function from the *statmod *package in R (http://www.r-project.org), which implements an exact binomial test to test for differential expression in individual genes. For the TMM method, we used the normalization constant to adjust library sizes in the sage.test function. We scaled RPKM values for each gene and library by a constant, such that the sum of all RPKM values equaled the original read total from both libraries. This scaling was necessary to compare significance values between the two read count methods, as the magnitude of the binomial test statistic is influenced by sample size. P-values from each method were then subjected to Benjamini-Hochberg correction for multiple testing [85] using the p.adjust function from the same package. We scored candidate genes with a corrected p-value < 0.001as DE.

Genes that are unique to a photoperiod treatment have an undefined log fold-change and log abundance, but these genes may be of particular biological importance. To include these genes in our calculations of fold-change, we added a value of 0.1 to the read count of all genes in each library. Fold-change values were calculated as log_2_(DI/*N*(DI)) - log_2_(NDI/*N*(NDI)), where *N*(DI) and *N*(NDI) are the normalized library sizes of the DI and NDI libraries, respectively.

### Verification of DE ESTs by qPCR

We used quantitative RT-PCR to verify the expression of candidate genes from two general categories: genes classified as over-expressed in the NDI and DI treatments from the TMM-normalized 454 analysis, and genes that were of *a priori *interest based on their role in the diapause response in other insects (See additional file 6: Results of qPCR validation). Previous studies on the physiological and molecular changes during diapause have revealed common themes of the diapause program across insects such as DNA replication and transcription, endocrine signaling, metabolism, and response to stress [5,8,9,21]. We used these categories to guide our choice of genes to test for DE using qPCR, because we were interested in determining whether diapause induction in *Ae. albopictus *showed commonalities of gene expression in genes putatively involved in these categories. We selected several genes from these categories, regardless whether they were significantly differentially expressed in the 454 dataset (See additional file 6: Results of qPCR validation).

Total RNA for qPCR was isolated from oocytes as described above. RNA pellets were stored at -70°C in 75% ethanol until they were used for cDNA synthesis. The concentration of resuspended RNA was measured using a Nano Drop spectrophotometer (Thermo Scientific, Wilmington DE USA). cDNA was synthesized using the iScript™ cDNA synthesis kit (Bio-Rad Laboratories, Inc. Hercules CA USA) according to the manufacturer''s instructions. One microgram of RNA was used in each synthesis reaction. To reduce variation caused by differences in the efficiency of the reverse transcription reaction, duplicate reactions were performed for each biological replicate and the products of the duplicate reactions were pooled then diluted before being used in qPCR reactions.

Relative mRNA abundance of selected genes of interest was measured using an iQ5™ Multicolor Real-Time PCR Detection System (Bio-Rad) and iQ™ SYBR Green Supermix (Bio-Rad). Each 20 μl reaction included 300 - 900 nM of the appropriate forward and reverse primers and 2 μl of cDNA template. PrimerQuest software (IDT DNA, Coralville, IA, USA) was used to design primer sequences which conform to MIQE standards (primer sequences available on request from JAR). Cycling parameters were 95°C for 3 min followed by 40-50 cycles of 95° for 10 s, 58° C for 30 s and 72° C for 30 s. Melt curve analysis and 1% agarose gel electrophoresis of PCR products verified that only one product was amplified in each reaction.

mRNA abundance was evaluated in 5 biological replicates for each group with three technical replicates for each primer pair. A modified 2^-ΔΔCt ^method [86] was used to calculate mRNA abundance for each gene of interest. Briefly, after averaging the threshold cycles (C_t _) of the technical replicates for each biological replicate, the geometric mean Ct for three reference genes, *RpL34, Histone H3*, and *Nucleosome Assembly Protein *(*NAP*), was subtracted from the mean Ct for each gene of interest (ΔCt). This value was then transformed to give relative mRNA abundance (2^-ΔCt^). Fold-change was calculated by dividing the mean relative abundance of DI replicates by the mean relative abundance of NDI replicates, and then taking the logarithm (log_2_) of this ratio. Student''s t-test was used to test for significant differences in relative mRNA abundance between groups, with a false discovery rate analysis [85] applied to reduce the probability of type I errors due to multiple comparison testing.

### Verification of DE ESTs - Significance comparisons

To determine whether expression levels from the 454 dataset and qPCR experiments were correlated, and whether the normalization method of the 454 dataset affected these correlations, we analyzed correlations in expression levels between both the TMM and RPKM normalization methods (454 EST set) and average mRNA abundance (qPCR data, 2^-ΔCt^) using the *lm *function in the package *stats *in R (http://www.r-project.org).

To address why correlation coefficients between 454 expression and mRNA abundance differed by photoperiod treatment, we calculated coefficients of variation (CVs) of mRNA abundance (2^-ΔCt^) from the five replicates of the qPCR data for each gene and photoperiod treatment. We then used a paired Student''s t-test to determine whether CVs were significantly different between photoperiod treatments.

We evaluated the correspondence of significant expression of each normalization method with significant expression of the qPCR data. First, for each normalization method, genes that were DE based on 454 expression levels were deemed "positive", and those that were not DE "negative". True positives occurred when the qPCR analysis confirmed significant over-expression in the same direction, and false positives were called when the qPCR result for a "positive" gene was not significant, or significant in the opposite direction. True negatives were non-significant in both the 454 and qPCR analysis, whereas false negatives actually demonstrated differential expression in the qPCR analysis. We then calculated the sensitivity and specificity of each method, where sensitivity is the number of true positives divided by all positive calls, and specificity is the number of true negatives divided by all negative calls.

## Authors' contributions

MFP prepared insect tissues, generated the mRNA for sequencing libraries and qPCR experiments, performed transcriptome assembly and sequence analysis, and drafted the manuscript. JAR designed and performed all qPCR experiments and helped to draft the manuscript. DLD participated in the design and coordination of the study and helped to draft the manuscript. CGE participated in the design and analysis of the study, contributed computational resources, and helped to draft the manuscript. PAA conceived of the study, participated in its design and coordination, and helped to draft the manuscript. All authors read and approved the final manuscript.

## Supplementary Material

Additional file 1 **Distribution of the number of ESTs assigned to a reference gene**. The x-axis is truncated at 100 EST matches per gene; 29 genes with 100-5,613 ESTs assigned to them are not included on the graph. Click here for file

Additional file 2 **Box plot of the percent length of each putative homolog matched by its *Ae. albopictus *EST**. Only ESTs from the "final" EST set are included. Reference length coverage values, which are calculated as the alignment length from the BLAST match, divided by the transcript length of the putative homolog. are displayed for each reference organism. *Ae. aegypti *genomic matches are not shown, as they encompass large stretches of non-coding sequence, and therefore the percent of the reference matched is unclear. Box plot symbols as in Figure 3. Click here for file

Additional file 3 **Box-plot of percent identities from BLAST alignments of final ESTs to each reference organism**. Box plot symbols as in Figure 3. Click here for file

Additional file 4 **Gene accumulation curve of *Ae. albopictus *ESTs**. The average cumulative number of recovered *Ae. aegypti *transcripts, plotted against the number of reads needed to obtain that number. All reads were searched against *Ae. aegypti *transcripts via *blastn*. The BLAST output order was randomized 1,000 times, and the average number of transcripts discovered with each additional read was calculated. Click here for file

Additional file 5 **Characteristics of all differentially expressed genes from the 454 dataset**. Gene ontology assignment, BLAST alignment, and expression statistics for the putative homolog of each differentially expressed EST. Click here for file

Additional file 6 **Results of qPCR validation**. Experimental details and results of qPCR for genes chosen for qPCR validation based on 454 differential expression or a priori expectation from previous diapause studies. Both putative functional information from the gene's homolog, as well as reaction conditions from the qPCR experiments, are shown. Fold-change values for qPCR experiments are given as log2(DI-NDI), where positive values indicate higher expression under DI conditions, and negative values indicate higher expression under NDI conditions. Click here for file

Additional file 7 **Gene IDs of *Ae. albopictus *rRNA and *Wolbachia *spp. used to pre-screen reads**. Click here for file

Additional file 8 **MIRA command line used for transcriptome assembly**. MIRA commands used to perform *de novo *transcriptome assembly on *Ae. albopictus *oocyte cDNA libraries generated under diapause-inducing and non-diapause-inducing photoperiods. Click here for file

## References

[B1] Tauber MJ Tauber CA Masaki S Seasonal adaptations of insects 1986 New York: Oxford University Press

[B2] Benoit J Denlinger D Suppression of water loss during adult diapause in the northern house mosquito, Culex pipiens Journal of Experimental Biology 2007 210 217 226 10.1242/jeb.02630 17210959

[B3] Hahn D Denlinger D Meeting the energetic demands of insect diapause: Nutrient storage and utilization Journal of Insect Physiology 2007 760 773 10.1016/j.jinsphys.2007.03.018 17532002

[B4] Hahn D Ragland G Shoemaker D Denlinger D Gene discovery using massively parallel pyrosequencing to develop ESTs for the flesh fly *Sarcophaga crassipalpis* BMC Genomics 2009 10 234 10.1186/1471-2164-10-234 19454017 PMC2700817

[B5] Ragland G Denlinger D Hahn D Mechanisms of suspended animation are revealed by transcript profiling of diapause in the flesh fly Proceedings of the National Academy of Sciences of the United States of America 2010 14909 14914 10.1073/pnas.1007075107 PMC2930464 20668242

[B6] Rinehart J Li A Yocum G Robich R Hayward S Denlinger D Up-regulation of heat shock proteins is essentail for cold survival during insect diapause Proceedings of the National Academy of Sciences of the United States of America 2007 104 11130 11137 10.1073/pnas.0703538104 17522254 PMC2040864

[B7] Yoder J Denlinger D Water-balance in flesh fly pupae and water-vapor absorption associated with diapause Journal of Experimental Biology 1991 157 273 286

[B8] Hahn D Denlinger D Energetics of Insect Diapause Annual Review of Entomology 2011 56 103 121 10.1146/annurev-ento-112408-085436 20690828

[B9] Denlinger DL Regulation of diapause Annual Review of Entomology 2002 93 122 10.1146/annurev.ento.47.091201.145137 11729070

[B10] Emerson K Uyemura A McDaniel K Schmidt P Bradshaw W Holzapfel C Environmental control of ovarian dormancy in natural populations of *Drosophila melanogaster* Journal of Comparative Physiology A 2009 195 825 829 10.1007/s00359-009-0460-5 19669646

[B11] MacRae TH Gene expression, metabolic regulation and stress tolerance during diapause Cellular and Molecular Life Sciences 2010 67 2405 2424 10.1007/s00018-010-0311-0 20213274 PMC11115916

[B12] Xu WH Sato Y Ikeda M Yamashita O Molecular characterization of the gene encoding the precursor protein of diapause hormone and pheromone biosynthesis activating neuropeptide (DH-PBAN) of the silkworm, *Bombyx mori *and its distribution in some insects Biochimica Biophysica Acta 1995 1261 83 89 10.1016/0167-4781(94)00238-x 7893764

[B13] Ekblom R Galindo J Applications of next generation sequencing in molecular ecology of non-model organisms Heredity 2011 107 1 15 10.1038/hdy.2010.152 21139633 PMC3186121

[B14] Wheat CW Fescemyer HW Kvist J Tas E Vera JC Frilander MJ Hanski I Marden JH Functional genomics of life history variation in a butterfly metapopulation Molecular Ecology 2011 20 1813 1828 10.1111/j.1365-294X.2011.05062.x 21410806

[B15] Emerson K Bradshaw W Holzapfel C Microarrays Reveal Early Transcriptional Events during the Termination of Larval Diapause in Natural Populations of the Mosquito, *Wyeomyia smithii* PLoS One 2010 5 e9574 10.1371/journal.pone.0009574 20221437 PMC2832704

[B16] Schwarz D Robertson HM Feder JL Varala K Hudson ME Ragland GJ Hahn DA Berlocher SH Sympatric ecological speciation meets pyrosequencing: sampling the transcriptome of the apple maggot *Rhagoletis pomonella* BMC Genomics 2009 10 633 10.1186/1471-2164-10-633 20035631 PMC2807884

[B17] Bao B Xu W Identification of gene expression changes associated with the initiation of diapause in the brain of the cotton bollworm, *Helicoverpa armigera* BMC Genomics 2011 12 224 10.1186/1471-2164-12-224 21569297 PMC3277317

[B18] Kostal V Eco-physiological phases of insect diapause Journal of Insect Physiology 2006 52 113 127 10.1016/j.jinsphys.2005.09.008 16332347

[B19] Urbanski J Aruda A Armbruster P A transcriptional element of the diapause program in the Asian tiger mosquito, *Aedes albopictus*, identified by suppressive subtractive hybridization Journal of Insect Physiology 2010 56 1147 1154 10.1016/j.jinsphys.2010.03.008 20230829

[B20] Urbanski J Benoit J Michaud M Denlinger D Armbruster P The molecular physiology of increased egg desiccation resistance during diapause in the invasive mosquito, *Aedes albopictus* Proceedings of the Royal Society B-Biological Sciences 2010 277 2683 2692 10.1098/rspb.2010.0362 PMC2982042 20410035

[B21] Reynolds J Hand S Embryonic diapause highlighted by differential expression of mRNAs for ecdysteroidogenesis, transcription and lipid sparing in the cricket *Allonemobius socius* Journal of Experimental Biology 2009 212 2074 2083 10.1242/jeb.027367 PMC2702454 19525434

[B22] Benedict M Levine R Hawley W Lounibos L Spread of the tiger: Global risk of invasion by the mosquito *Aedes albopictus* Vector-Borne and Zoonotic Diseases 2007 76 85 10.1089/vbz.2006.0562 PMC2212601 17417960

[B23] Lounibos L Invasions by insect vectors of human disease Annual Review of Entomology 2002 233 266 10.1146/annurev.ento.47.091201.145206 11729075

[B24] Mori A Oda T Wada Y Studies on the egg diapause and overwintering of *Aedes albopictus *in Nagasaki Tropical Medicine 1981 23 79 90

[B25] Wang RL Observations on the influence of photoperiod on egg diapause in *Aedes albopictus *Skuse Acta Entomologica Sinica 1966 15 75 77

[B26] Mogi M Okazawa T Sota T Geographical pattern in autogeny and wing length in *(Diptera: Cu*licidae) Mosquito Systematics 1995 27 155 166

[B27] Lounibos L Escher R Lourenco-de-Oliveria R Asymmetric evolution of photoperiodic diapause in temperate and tropical invasive populations of *Aedes albopictus *(Diptera: Culicidae) Annals of the Entomological Society of America 2003 96 512 518 10.1603/0013-8746(2003)096[0512:AEOPDI]2.0.CO;2

[B28] Denlinger DL Why study diapause? Entomological Research 2008 38 1 9 10.1111/j.1748-5967.2008.00139.x

[B29] Nene V Wortman JR Lawson D Haas B Kodira C Tu Z Loftus B Xi Z Megy K Grabherr M Genome Sequence of *Aedes aegypti*, a Major Arbovirus Vector Science 2007 316 1718 1723 10.1126/science.1138878 17510324 PMC2868357

[B30] Bettencourt R Pinheiro M Egas C Gomes P Afonso M Shank T Santos R High-throughput sequencing and analysis of the gill tissue transcriptome from the deep-sea hydrothermal vent mussel *Bathymodiolus azoricus* BMC Genomics 2010 11 559 10.1186/1471-2164-11-559 20937131 PMC3091708

[B31] Der J Barker M Wickett N dePamphilis C Wolf P De novo characterization of the gametophyte transcriptome in bracken fern, *Pteridium aquilinum* BMC Genomics 2011 12 99 21303537 10.1186/1471-2164-12-99 PMC3042945

[B32] Coppe A Pujolar J Maes G Larsen P Hansen M Bernatchez L Zane L Bortoluzzi S Sequencing, *de novo *annotation and analysis of the first *Anguilla anguilla *transcriptome: EeelBase opens new perspectives for the study of the critically endangered european eel BMC Genomics 2010 11 635 10.1186/1471-2164-11-635 21080939 PMC3012609

[B33] Zagrobelny M Scheibye-Alsing K Jensen N Moller B Gorodkin J Bak S 454 pyrosequencing based transcriptome analysis of *Zygaena filipendulae *with focus on genes involved in biosynthesis of cyanogenic glucosides BMC Genomics 2009 10 574 10.1186/1471-2164-10-574 19954531 PMC2791780

[B34] Chevreux B Pfisterer T Drescher B Driesel A Muller W Wetter T Suhai S Using the miraEST assembler for reliable and automated mRNA transcript assembly and SNP detection in sequenced ESTs Genome Research 2004 14 1147 1159 10.1101/gr.1917404 15140833 PMC419793

[B35] Kumar S Blaxter M Comparing *de novo *assemblers for 454 transcriptome data BMC Genomics 2010 11 571 10.1186/1471-2164-11-571 20950480 PMC3091720

[B36] Choi J Kijimoto T Snell-Rood E Tae H Yang Y Moczek A Andrews J Gene discovery in the horned beetle *Onthophagus taurus* BMC Genomics 2010 11 703 10.1186/1471-2164-11-703 21156066 PMC3019233

[B37] Parchman T Geist K Grahnen J Benkman C Buerkle C Transcriptome sequencing in an ecologically important tree species: assembly, annotation, and marker discovery BMC Genomics 2010 11 180 10.1186/1471-2164-11-180 20233449 PMC2851599

[B38] Ferguson L Lee S Chamberlain N Nadeau N Joron M Baxter S Wilkinson P Papanicolaou A Kumar S Kee T Characterization of a hotspot for mimicry: assembly of a butterfly wing transcriptome to genomic sequence at the HmYb/Sb locus Molecular Ecology 2010 19 240 254 20331783 10.1111/j.1365-294X.2009.04475.x

[B39] Altschul SF Gish W Miller W Myers EW Lipman DJ Basic local alignment search tool Journal of Molecular Biology 1990 215 403 410 2231712 10.1016/S0022-2836(05)80360-2

[B40] Papanicolaou A Stierli R Ffrench-Constant R Heckel D Next generation transcriptomes for next generation genomes using est2assembly BMC Bioinformatics 2009 10 447 10.1186/1471-2105-10-447 20034392 PMC3087352

[B41] Crawford J Guelbeogo W Sanou A Traore A Vernick K Sagnon N Lazzaro B *De Novo *Transcriptome Sequencing in *Anopheles funestus *Using Illumina RNA-Seq Technology PLoS ONE 2010 5 e14202 10.1371/journal.pone.0014202 21151993 PMC2996306

[B42] Gregory R Darby A Irving H Coulibaly M Hughes M Koekemoer L Coetzee M Ranson H Hemingway J Hall N Wondji C A *De Novo *Expression Profiling of *Anopheles funestus*, Malaria Vector in Africa, Using 454 Pyrosequencing PLoS ONE 2011 6 e17418 10.1371/journal.pone.0017418 21364769 PMC3045460

[B43] McIntyre L Lopiano K Morse A Amin V Oberg A Young L Nuzhdin S RNA-seq: technical variability and sampling BMC Genomics 2011 12 293 10.1186/1471-2164-12-293 21645359 PMC3141664

[B44] Robinson M Oshlack A A scaling normalization method for differential expression analysis of RNA-seq data Genome Biology 2010 11 R25 10.1186/gb-2010-11-3-r25 20196867 PMC2864565

[B45] Mortazavi A Williams BA McCue K Schaeffer L Wold B Mapping and quantifying mammalian transcriptomes by RNA-Seq Nature Methods 2008 5 621 628 10.1038/nmeth.1226 18516045 PMC13303166

[B46] Roberts A Trapnell C Donaghey J Rinn JL Pachter L Improving RNA-Seq expression estimates by correcting for fragment bias Genome Biology 2011 12 10.1186/gb-2011-12-3-r22 PMC3129672 21410973

[B47] Fleige S Pfaffl M RNA integrity and the effect on the real time qRT-PCR performance Molecular Aspects of Medicine 2006 27 126 139 10.1016/j.mam.2005.12.003 16469371

[B48] Feng X Hara Y Riabowol K Different HATS of the ING1 gene family Trends in Cell Biology 2002 12 532 538 10.1016/S0962-8924(02)02391-7 12446115

[B49] Lunardia A Di Minina G Proveroc P Dal Ferroa M Carottia M Del Sala G Collavina L A genome-scale protein interaction profile of *Drosophila *p53 uncovers additional nodes of the human p53 network Proceedings of the National Academy of Sciences of the United States of America 2010 107 6322 6327 10.1073/pnas.1002447107 20308539 PMC2851947

[B50] Alic N Hoddinott MP Vinti G Partridge L Lifespan extension by increased expression of the *Drosophila *homologue of the IGFBP7 tumour suppressor Aging Cell 2011 10 137 147 10.1111/j.1474-9726.2010.00653.x 21108726 PMC3042147

[B51] Sans C Satterwhite D Stoltzman C Breen K Ayer D MondoA-Mlx heterodimers are candidate sensors of cellular energy status: Mitochondrial localization and direct regulation of glycolysis Molecular and Cellular Biology 2006 26 4863 4871 10.1128/MCB.00657-05 16782875 PMC1489152

[B52] Sim C Denlinger DL Insulin signaling and FOXO regulate the overwintering diapause of the mosquito *Culex pipiens* Proceedings of the National Academy of Sciences of the United States of America 2008 105 6777 6781 10.1073/pnas.0802067105 18448677 PMC2373331

[B53] Denlinger DL Yocum GD Rinehart JP Gilbert LI Hormonal control of diapause Insect Endocrinology 2012 London, UK: Academic Press 430 463

[B54] Lee K Denlinger D A role for ecdysteroids in the induction and maintenance of the pharate first instar diapause of the gypsy moth, Lymantria dispar Journal of Insect Physiology 1997 43 289 296 10.1016/S0022-1910(96)00082-0 12769913

[B55] Kadrmas JL Smith MA Pronovost SM Beckerle MC Characterization of RACK1 function in *Drosophila *development Developmental Dynamics 2007 236 2207 2215 10.1002/dvdy.21217 17584887

[B56] Quan G Krell P Arif B Feng Q Receptor of activated C kinase 1 (RACK1) is necessary for the 20-hydroxyecdysone-induced expression of the transcription factor CHR3 in the spruce budworm *Choristoneura fumiferana* Insect Molecular Biology 2006 15 79 87 10.1111/j.1365-2583.2006.00611.x 16469071

[B57] Robles M Boyault C Knutti D Padmanabhan K Weitz C Identification of RACK1 and Protein Kinase C alpha as Integral Components of the Mammalian Circadian Clock Science 2010 327 463 466 10.1126/science.1180067 20093473

[B58] Bunning E Die endonome Tagesrhythmik als Grundlage der photoperiodischen Reaktion Bericht Deutscher Botanischen Gesellschaft 1936 54 590 607

[B59] Ikeno T Tanaka SI Numata H Goto SG Photoperiodic diapause under the control of circadian clock genes in an insect BMC Biology 2010 8 116 10.1186/1741-7007-8-116 20815865 PMC2942818

[B60] Tauber E Zordan M Sandrelli F Pegoraro M Osterwalder N Breda C Daga A Selmin A Monger K Benna C Natural selection favors a newly derived timeless allele in *Drosophila melanogaster* Science 2007 316 1895 1898 10.1126/science.1138412 17600215

[B61] Bradshaw WE Holzapfel CM Circadian clock genes, ovarian development and diapause BMC Biology 2010 8 115 10.1186/1741-7007-8-115 20828372 PMC2933584

[B62] Garbe JC Yang E Fristrom JW IMP-L2: an essential secreted immunoglobulin family member implicated in neural and ectodermal development in *Drosophila* Development 1993 119 1237 1250 8306886 10.1242/dev.119.4.1237

[B63] Honegger B Galic M Köhler K Wittwer F Brogiolo W Hafen E Stocker H Imp-L2, a putative homolog of vertebrate IGF-binding protein 7, counteracts insulin signaling in *Drosophila *and is essential for starvation resistance Journal of Biology 2008 7 10 10.1186/jbiol72 18412985 PMC2323038

[B64] Tennessen J Thummel C Coordinating Growth and Maturation - Insights from Drosophila Current Biology 2011 21 R750 R757 10.1016/j.cub.2011.06.033 21959165 PMC4353487

[B65] Lin Y-J Seroude L Benzer S Extended Life-Span and Stress Resistance in the *Drosophila *Mutant *methuselah* Science 1998 282 943 946 9794765 10.1126/science.282.5390.943

[B66] Schmidt PS Duvernell DD Eanes WF Adaptive evolution of a candidate gene for aging in *Drosophila* Proceedings of the National Academy of Sciences of the United States of America 2000 97 10861 10865 10995474 10.1073/pnas.190338897 PMC27114

[B67] Paaby AB Schmidt PS Functional Significance of Allelic Variation at *methuselah*, an Aging Gene in *Drosophila* PLoS ONE 2008 3 e1987 10.1371/journal.pone.0001987 18414670 PMC2288678

[B68] Fielenbach N Antebi A *C. elegans *dauer formation and the molecular basis of plasticity Genes & Development 2008 22 2149 2165 10.1101/gad.1701508 18708575 PMC2735354

[B69] Homma T Watanabe K Tsurumaru S Kataoka H Imai K Kamba M Niimi T Yamashita O Yaginuma T G protein-coupled receptor for diapause hormone, an inducer of *Bombyx *embryonic diapause Biochemical and Biophysical Research Communications 2006 344 386 393 10.1016/j.bbrc.2006.03.085 16600181

[B70] Kikuchi R Kawahigashi H Ando T Tonooka T Handa H Molecular and Functional Characterization of PEBP Genes in Barley Reveal the Diversification of Their Roles in Flowering Plant Physiology 2009 149 1341 1353 10.1104/pp.108.132134 19168644 PMC2649388

[B71] Gyllenstrand N Clapham D Källman T Lagercrantz U A Norway Spruce FLOWERING LOCUS T Homolog Is Implicated in Control of Growth Rhythm in Conifers Plant Physiology 2007 144 248 257 10.1104/pp.107.095802 17369429 PMC1913773

[B72] Armbruster P Hutchinson R Pupal mass and wing length as indicators of fecundity in *Aedes albopictus *and *Aedes geniculatus *(Diptera: Culicidae) Journal of Medical Entomology 2002 699 704 10.1603/0022-2585-39.4.699 12144308

[B73] Armbruster P Conn J Geographic variation of larval growth in North American *Aedes albopictus *(Diptera: Culicidae) Annals of the Entomological Society of America 2006 1234 1243

[B74] Clements AN The Biology of Mosquitoes: Development, Nutrition and Reproduction 1992 London, UK: Chapman and Hall

[B75] Novak JN Shroyer DA Eggs of *Aedes triseriatus *and *Aedes hendersoni: A *method to simulate optimal hatch Mosquito News 1978 38 515 521

[B76] Trpis M A new bleaching and decalcifying method for general use in zoology Canadian Journal of Zoology 1970 48 892 893 10.1139/z70-158

[B77] Hawley W Pumpuni C Brady R Craig G Overwintering survival of *Aedes albopictus *(Diptera, Culicidae) eggs in Indiana Journal of Medical Entomology 1989 26 122 129 2709388 10.1093/jmedent/26.2.122

[B78] Huse SM Huber JA Morrison HG Sogin ML Welch DM Accuracy and quality of massively parallel DNA pyrosequencing Genome Biology 2007 8 R143 10.1186/gb-2007-8-7-r143 17659080 PMC2323236

[B79] Gomez-Alvarez V Teal T Schmidt T Systematic artifacts in metagenomes from complex microbial communities Isme Journal 2009 1314 1317 10.1038/ismej.2009.72 19587772

[B80] Dlugosch KM Rieseberg LH SnoWhite: A pipeline for aggressive cleaning of next-generation sequence reads http://www.evopipes.net

[B81] Ning Z Cox A Mullikin J SSAHA: A fast search method for large DNA databases Genome Research 2001 1725 1729 10.1101/gr.194201 PMC311141 11591649

[B82] Margulies M Egholm M Altman WE Attiya S Bader JS Bemben LA Berka J Braverman MS Chen YJ Chen Z Genome sequencing in microfabricated high-density picolitre reactors Nature 2005 437 376 380 16056220 10.1038/nature03959 PMC1464427

[B83] Vera J Wheat C Fescemyer H Frilander M Crawford D Hanski I Marden J Rapid transcriptome characterization for a nonmodel organism using 454 pyrosequencing Molecular Ecology 2008 1636 1647 10.1111/j.1365-294X.2008.03666.x 18266620

[B84] Marioni JC Mason CE Mane SM Stephens M Gilad Y RNA-seq: An assessment of technical reproducibility and comparison with gene expression arrays Genome Research 2008 18 1509 1517 10.1101/gr.079558.108 18550803 PMC2527709

[B85] Benjamini Y Hochberg Y Controlling the false discovery rate: a practical and powerful approach to multiple testing Journal of the Royal Statistical Society Series B (Methodological) 1995 57 289 300

[B86] Livak KJ Schmittgen TD Analysis of relative gene expression data using real-time quantitative PCR and the 2(-Delta Delta C(T)) Method Methods 2001 25 United States: 2001 Elsevier Science (USA) 402 408 10.1006/meth.2001.1262 11846609

