# Supplementary figures and images for: A de novo transcriptome of the Asian tiger mosquito, Aedes albopictus, to identify candidate transcripts for diapause preparation
Source: BMC Genomics. 2011 Dec 20;12:619. doi: 10.1186/1471-2164-12-619 (PMC3258294; doi:10.1186/1471-2164-12-619)

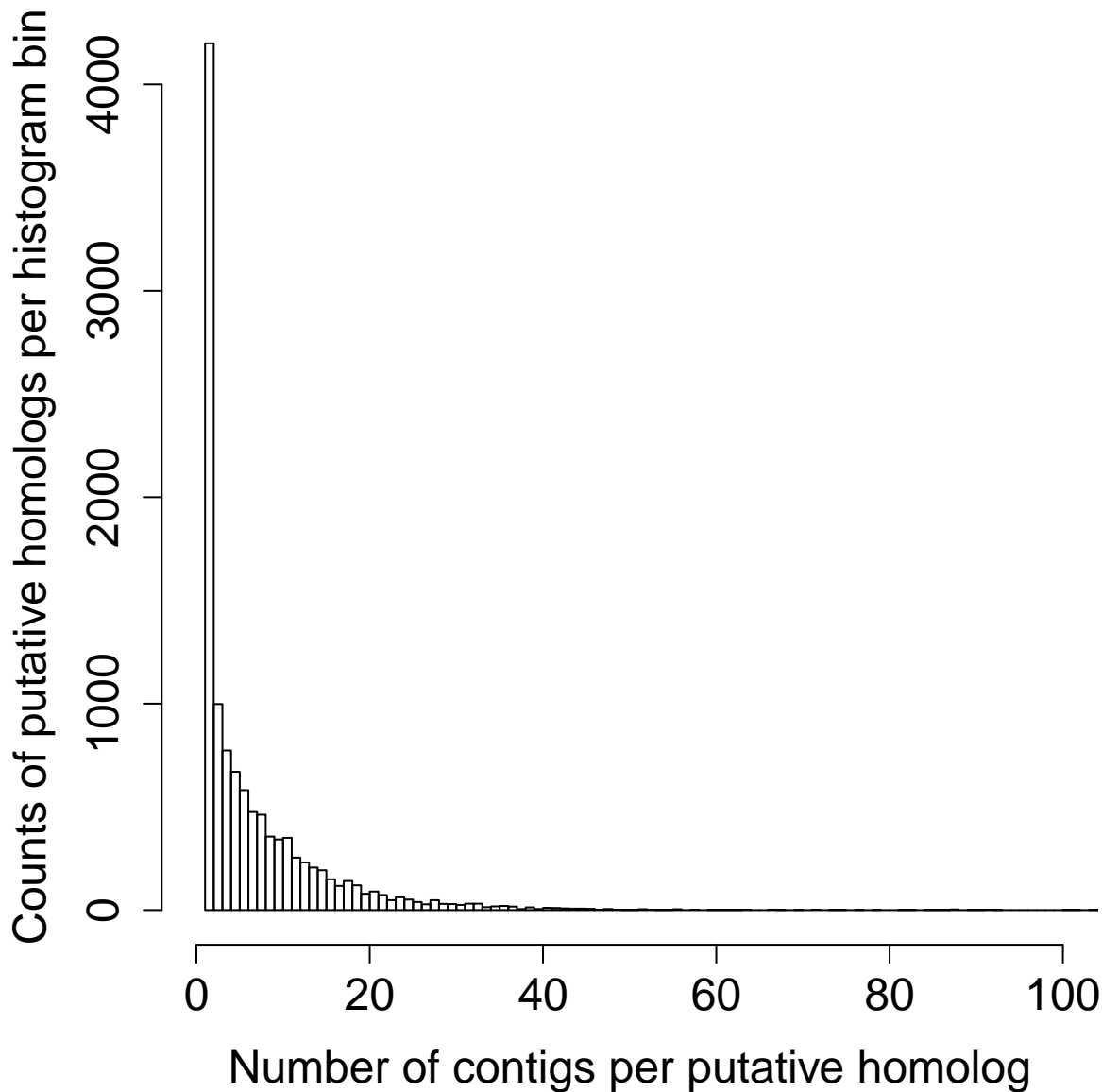

Supplement: Additional file 1 — Distribution of the number of ESTs assigned to a reference gene. The x-axis is truncated at 100 EST matches per gene; 29 genes with 100-5,613 ESTs assigned to them are not included on the graph. [file 1471-2164-12-619-S1.PDF]

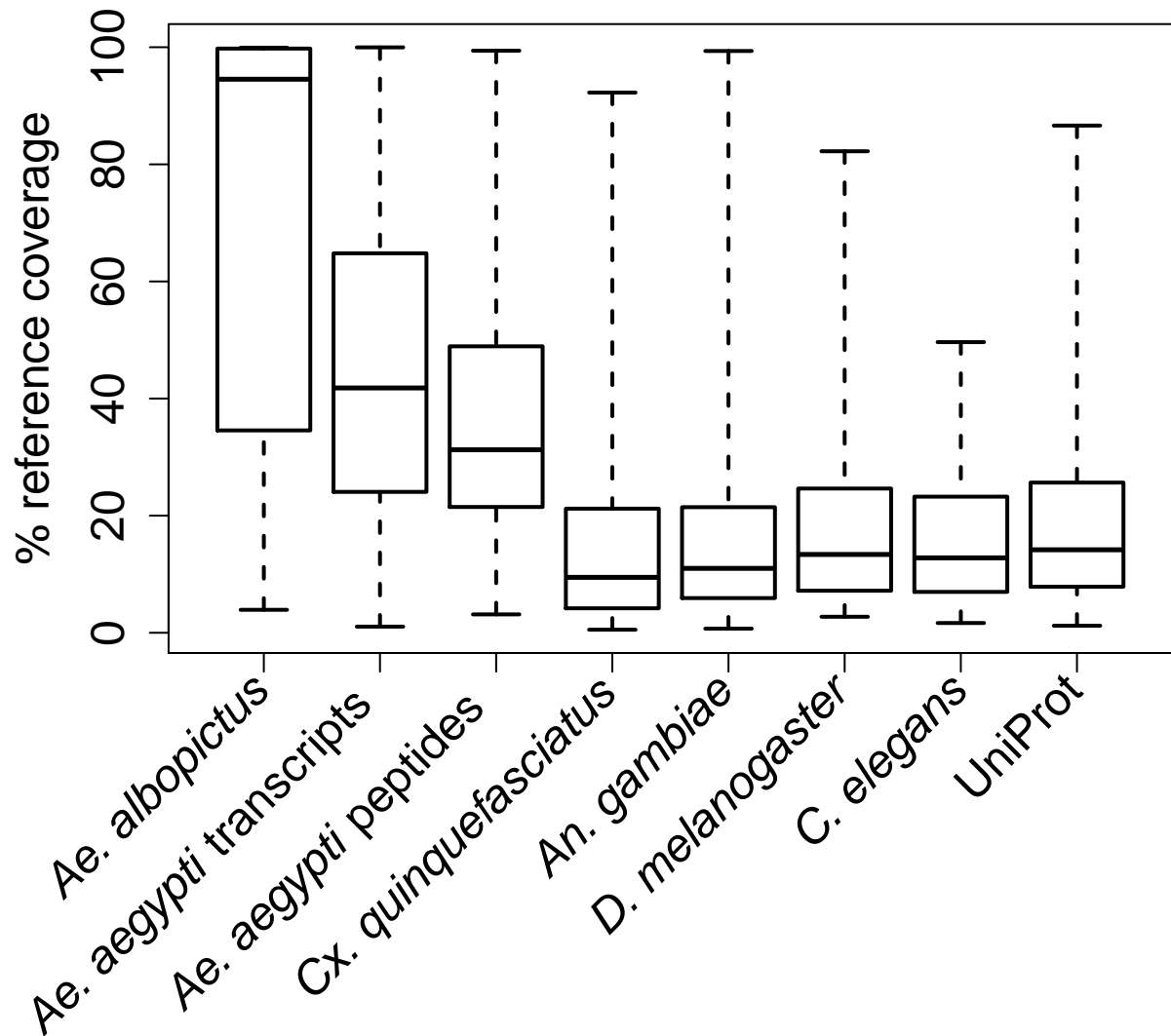

Supplement: Additional file 2 — Box plot of the percent length of each putative homolog matched by its Ae. albopictus EST. Only ESTs from the "final" EST set are included. Reference length coverage values, which are calculated as the alignment length from the BLAST match, divided by the transcript length of the putative homolog. are displayed for each reference organism. Ae. aegypti genomic matches are not shown, as they encompass large stretches of non-coding sequence, and therefore the percent of the reference matched is unclear. Box plot symbols as in Figure 3. [file 1471-2164-12-619-S2.PDF]

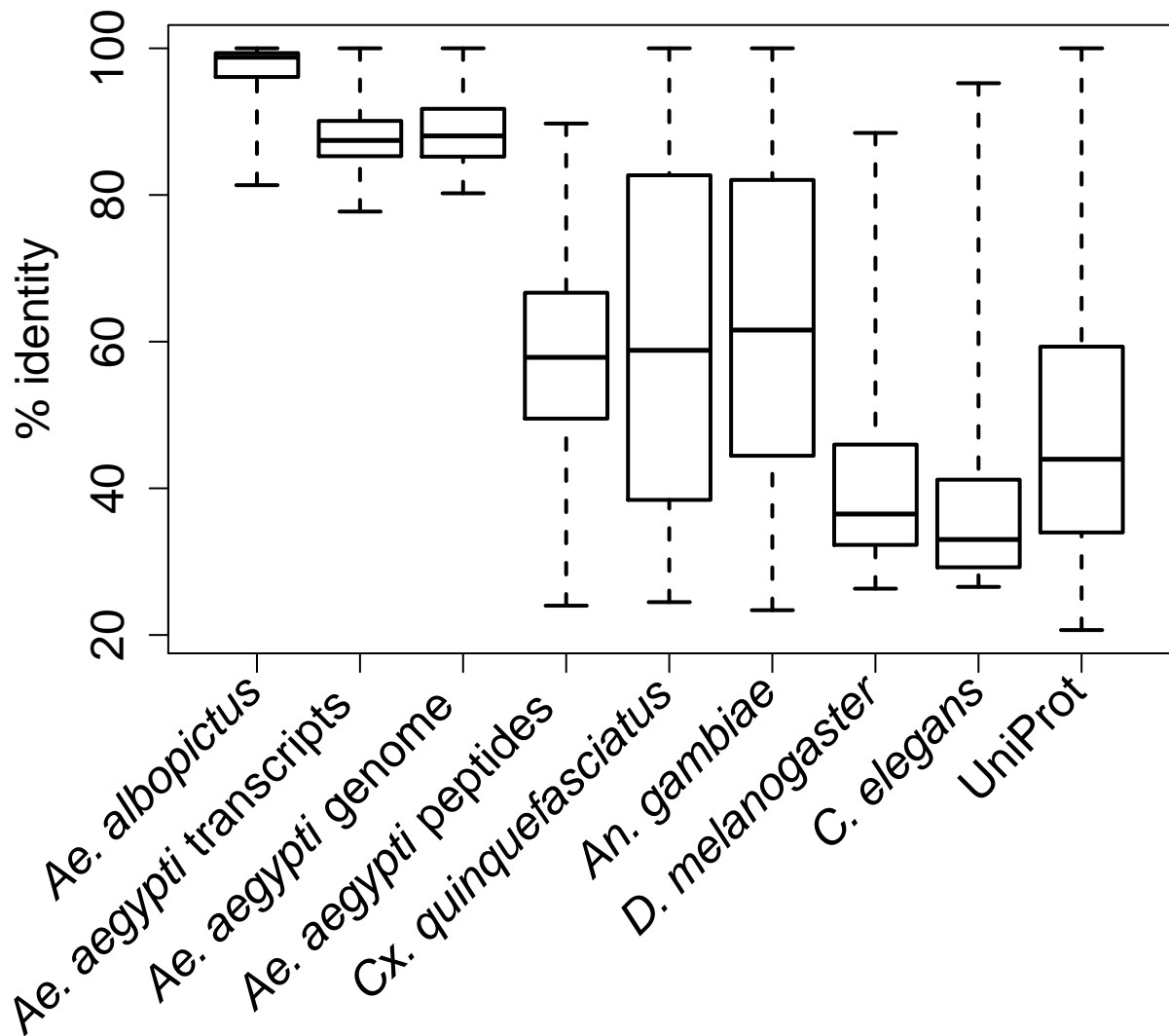

Supplement: Additional file 3 — Box-plot of percent identities from BLAST alignments of final ESTs to each reference organism. Box plot symbols as in Figure 3. [file 1471-2164-12-619-S3.PDF]

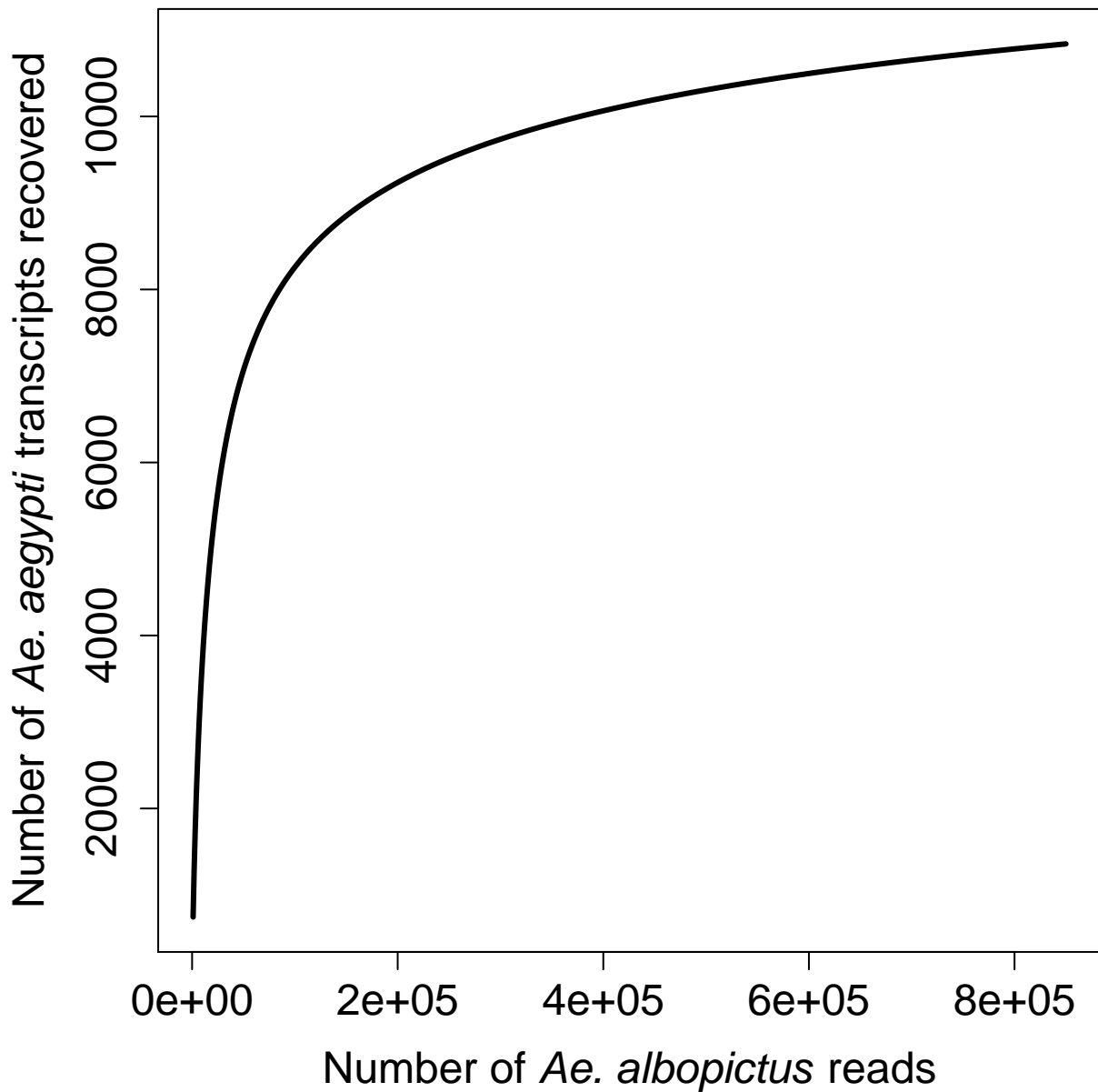

Supplement: Additional file 4 — Gene accumulation curve of Ae. albopictus ESTs. The average cumulative number of recovered Ae. aegypti transcripts, plotted against the number of reads needed to obtain that number. All reads were searched against Ae. aegypti transcripts via blastn. The BLAST output order was randomized 1,000 times, and the average number of transcripts discovered with each additional read was calculated. [file 1471-2164-12-619-S4.PDF]
