# Supplementary material for: A de novo transcriptome of the Asian tiger mosquito, Aedes albopictus, to identify candidate transcripts for diapause preparation
Source: BMC Genomics. 2011 Dec 20;12:619. doi: 10.1186/1471-2164-12-619 (PMC3258294; doi:10.1186/1471-2164-12-619)
Supplement: Additional file 8 — MIRA command line used for transcriptome assembly. MIRA commands used to perform de novo transcriptome assembly on Ae. albopictus oocyte cDNA libraries generated under diapause-inducing and non-diapause-inducing photoperiods. [file 1471-2164-12-619-S8.DOC]

**Additional File 7.** MIRA commands used to perform *de novo* transcriptome assembly on *Ae. albopictus* oocyte cDNA libraries generated under diapause-inducing and non-diapause-inducing photoperiods.

mira --project=all_new --job=denovo,est,normal,454 -FN:svsi=all_new_ssahavectorscreen_in.txt -AS:nop=3 -SK:not=6:bph=15:mnr=yes:mmhr=1 -CO:asir=yes 454_SETTINGS -CL:msvs=1:msvsmfg=5:msvsmeg=5 -AS:ardct:3.0 -LR:mxti=no -AL:mo=38 -ED:ace=1
